# Association of Overcrowding and Turnover with Self-Harm in a Swiss Pre-Trial Prison

**DOI:** 10.3390/ijerph15040601

**Published:** 2018-03-27

**Authors:** Stéphanie Baggio, Laurent Gétaz, Nguyen Toan Tran, Nicolas Peigné, Komal Chacowry Pala, Diane Golay, Patrick Heller, Patrick Bodenmann, Hans Wolff

**Affiliations:** 1 Division of Prison Health, Geneva University Hospitals & University of Geneva, 1225 Geneva, Switzerland; laurent.getaz@hcuge.ch (L.G.); nguyen-toan.tran@hcuge.ch (N.T.T.); nicolas.peigne@hcuge.ch (N.P.); komal.r.chacowry@hcuge.ch (K.C.P.); diane.roth-golay@hcuge.ch (D.G.); patrick.heller@hcuge.ch (P.H.); hans.wolff@hcuge.ch (H.W.); 2 Life Course and Inequality Research Centre, University of Lausanne, 1015 Lausanne, Switzerland; 3 Australian Centre for Public Population Health Research, Faculty of Health, University of Technology, 2007 Sydney, Australia; 4 Vulnerable Population Center, Department of Ambulatory Care and Community Medicine, Lausanne University Hospital & University of Lausanne, 1011 Lausanne, Switzerland; patrick.bodenmann@hospvd.ch

**Keywords:** custody, health care, prevention, prisoners, suicide

## Abstract

Self-harm is a common issue in detention and includes both suicidal and non-suicidal behaviours. Beyond well-known individual risk factors, institutional factors such as overcrowding (i.e., when the prison population exceeds its capacity) and turnover (i.e., the rate at which the prison population is renewed), may also increase the risk of self-harm. However, these factors are understudied or previous studies reported inconsistent findings. This study investigated the association of self-harm with overcrowding and turnover in the largest pre-trial Swiss prison in Geneva. Data were collected yearly between 2011 and 2017. Measures included self-harm (all kinds of self-injuring acts requiring medical attention, including self-strangulations and self-hangings). We performed meta-regressions to analyse the relationships between self-harm and institutional factors. Self-harm events were frequent, with a prevalence estimate of 26.4%. Overcrowding and turnover were high (average occupation rate of 177% and average turnover of 73%, respectively). Overcrowding and turnover were significantly associated with self-harm (respectively b = 0.068, *p* < 0.001 and (b = 1.257, *p* < 0.001). In both cases, self-harm was higher when overcrowding and turnover increased. Overcrowding and turnover raise important human rights concerns and have damaging effects on the health of people living in detention. Identification of and care for this vulnerable population at risk of self-harm are needed and institutional factors should be addressed.

## 1. Introduction

Self-harm, which includes both suicidal and non-suicidal behaviours, is a major problem in detention settings worldwide. Self-harm is associated with specific well-known individual risk factors, such as psychiatric disorders [1,2]. Institutional factors may also increase the risk of self-harm [3,4]. Recently, attention has been drawn to prison overcrowding, that is, when the prison population (occupation rate) exceeds the official prison capacity [5,6,7,8,9,10,11]. Overcrowding results in a lack of basic comfort and privacy which are essential in preserving a person’s dignity (e.g., sharing cells designed for one person). In addition, access to time outside the prison cell, vocational activities, physical exercise, or health services cannot be adequately ensured due to a lack of suitable infrastructure and human resources. All these factors are likely to increase the vulnerability of PLD (people living in detention), that is, risks for social, physical and psychological health of PLD [12]. Furthermore, overcrowding may increase the risk of self-harm and suicide among PLD [7,8,9,10,11]. However, previous studies investigating this association reported inconsistent findings. Overcrowding was associated with prison suicide in some studies [7,8,9,10] but not in others [5,6]. Overcrowding was associated with specific self-harm events (self-strangulations and self-hangings) in one study [11] but to our knowledge, no other studies focused on the relationship between overcrowding and non-suicidal self-injury (including self-injury without intent to die). Non-suicidal self-injury can lead to subsequent suicide [2] and, therefore, achieving a better understanding of the mechanisms affecting prison suicide is paramount. Given the inconsistent results and the lack of data, empirical studies on this topic are needed. Additionally, it would provide insights into the recent debate on the importance of prison-level factors in preventing suicide in prison [3,4].

Another important but neglected institutional factor is prison turnover. Turnover can be defined as the rate at which the prison population is renewed. A high prison turnover means that there is a large proportion of new PLD. Prison entry is associated with acute distress [13] and an increased risk of suicide [14]. Additionally, a high turnover may contribute to the instability of social relationships (e.g., ability to form networks and to develop supportive relationships with prison staff and other PLD [15]) and therefore may contribute to PLD’s psychological distress. Worldwide, many prisons face high turnover of PLD (on average 51.8% [16]) but the consequences of turnover are understudied. Few studies included prison turnover as a predictor of self-harm and they reported inconsistent findings. Turnover was significantly associated with prison suicide in one study [15] but not in another [5]. In van Ginneken et al.’s study [15], the association between overcrowding and suicide became non-significant when turnover was controlled for. To our knowledge, no study investigated the association between turnover and self-harm, including non-suicidal behaviours.

Therefore, this study aimed to fill the existing gaps in knowledge by investigating the association of self-harm with overcrowding and turnover in a Swiss prison, using observational data over a seven-year period. 

## 2. Materials and Methods 

Data were collected between 2011 and 2017 in the pre-trial prison of Champ-Dollon, which is located outside of Geneva, Switzerland. Champ-Dollon is the largest prison in Switzerland. The prison capacity is 376 since 2011 (with 22 additional places in 2017). Most PLD spend 23 h a day in their cell and have access to outdoor activities only for one h a day. 

Information on prison overcrowding and turnover for each year was obtained from the annual prison report. The annual overcrowding index was computed by dividing the annual mean daily population by the prison capacity. The annual turnover ratio for the whole prison was calculated as in Fazel et al. [1,2], using the number of releases divided by the number of admissions plus the average prison population of the previous year. Self-harm events were registered by nurses who are on location 24 h a day, seven days a week. They recorded each self-harm event requiring medical attention immediately after its occurrence. This information was available in a secure database in the prison medical unit. Self-harm included non-suicidal behaviours (self-injury) and suicidal ones (self-strangulation and self-hanging). We recorded the number of self-harm events for each year.

Ethical approval was not required because we used anonymized quality control data.

To test the relationship of self-harm with overcrowding and turnover, we used a fixed-effect multivariate meta-regression considering each year as a separate sample. The predictors were prison overcrowding and turnover and the outcome was the prevalence rate of self-harm events. We also provided the meta-analytic prevalence rates of self-harm and an overview of the overcrowding and turnover over the study period. Analyses were performed using R 3.4.3 (R Foundation for Statistical Computing, Vienna, Austria) and the package ‘metafor’ version 2.0.0 [17].

## 3. Results

**Figure 1:**
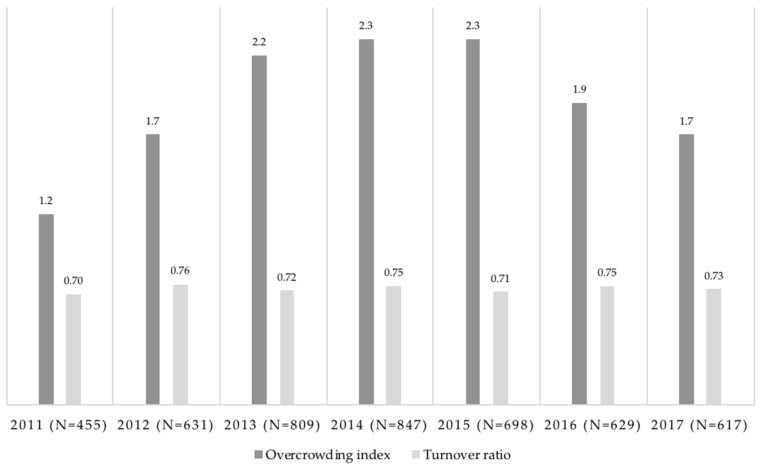
Descriptive statistics for overcrowding and turnover and number of PLD (people living in detention) each year.

The prevalence estimate of self-harm events was 26.4% (95% confidence interval (CI): 25.1–26.2). Descriptive statistics of overcrowding and turnover are reported in Figure 1. Overcrowding had an increasing trend until 2014, after which it started to decrease. On average, the overcrowding index was 1.77 over the seven-year period, which corresponded to an occupation rate of 177% above the official prison capacity. On average, the turnover was 73% and remained relatively stable during the seven-year period, which meant that 73% of the prison population was entirely renewed each year.

**Figure 2:**
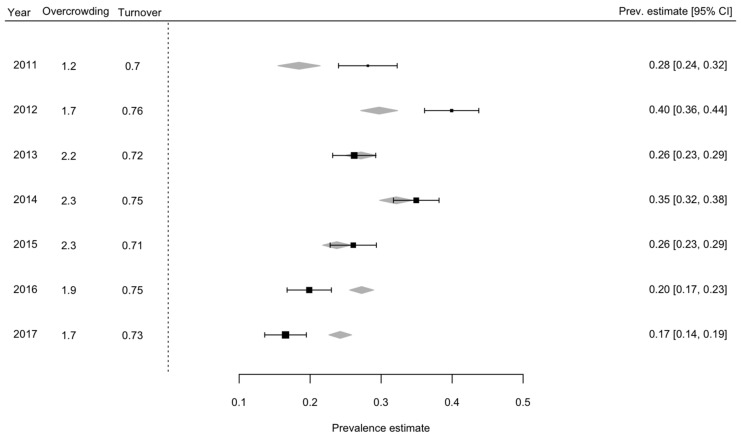
Forest plot of self-harm over seven years at the Champ-Dollon prison.

Results of the meta-regressions are reported in Table 1. Overcrowding was significantly associated with self-harm (b = 0.068, *p* < 0.001), independently from turnover. The prevalence of self-harm was higher when overcrowding increased. Turnover was significantly associated with self-harm (b = 1.266, *p* < 0.001) independently from overcrowding. A higher turnover was associated with an increased prevalence estimate of self-harm. Results of the meta-regression is shown in Figure 2.

The measure of effect is represented with a square, with 95% confidence intervals represented with horizontal lines. Diamonds represent global effect of all covariates included in the model (overcrowding and turnover).

## 4. Discussion

Overall, institutional prison factors of overcrowding and turnover were significantly associated with self-harm events in the Switzerland’s largest pre-trial prison. First, overcrowding was associated with an increased prevalence rate of self-harm events, including self-strangulation and self-hanging. This result is in line with previous studies [7,8,9,10,11] and contributes to the growing evidence that prison overcrowding has deleterious effects on PLD self-harm. Prison overcrowding is a worldwide concern with several countries having an occupation rate exceeding their capacity [15]. 

Second, turnover was also associated with an increased prevalence rate of self-harm events. This finding was consistent with van Ginneken et al.’s conclusions [15]. Turnover may increase self-harm and suicide because of a large range of reasons, including the large proportion of prison entries, which is a risky period for suicide [14] and instability in establishing and maintaining social relationships [15]. In our study, overcrowding was independently associated with self-harm events when turnover was controlled for. This meant that both these institutional factors are important, whereas van Ginneken et al. [15] concluded that overcrowding was not independently associated with prison suicide when turnover was controlled for. 

Overall, institutional factors appear to be important factors that are susceptible to increase harmful behaviours in prison [11]. As non-suicidal self-injury can lead to subsequent suicide [2], these results are also relevant for suicide research. Policies and preventive actions and interventions designed to reduce self-harm in prison should therefore not focus exclusively on individual-level factors. This result is in line with the recommendations of Bartlett et al. [3], which highlight the importance of addressing institutional factors. To our knowledge, this is the first study showing that overcrowding and turnover are associated with self-harm, including non-suicidal behaviours. 

Our preliminary findings highlight worrisome characteristics of pre-trial detention. First, there was a high prevalence estimate of self-harm events: there was more than a quarter of events needing medical attention because of self-injuring, self-strangulation, or self-hanging. This prevalence rate was higher than those reported in previous studies (UK: 5–6% in men [2]). Second, overcrowding and turnover were very high, respectively 177% and 73% on average over the seven-year period, which confirms the general situation of pre-trial prisons worldwide [16]. However, the reduction of self-harm events along with the decrease in the overcrowding index is a positive evolution for the Champ-Dollon prison. The stable turnover rate may be due to the fact that a notable proportion of PLD incarcerated in the prison were already sentenced, even if they were detained in a pre-trial prison. 

This study has some limitations. First, it was not possible to distinguish between self-strangulation and self-hanging and therefore classifying individuals as intending to die or not was not possible. Second, we recorded the number of self-harm events and the prevalence rate may have been influenced by some PLD who have several self-harm events over time. Some of these PLD were transferred to the forensic psychiatric unit Curabilis, opened in 2014. This may have reduced the number of repeated self-harm events after 2014. Another limitation was the lack of individual data, such as the prevalence of psychiatric disorders or other individual risk factors. Future studies should include a large range of individual factors to provide a general idea of the importance of institutional factors in comparison with individual factors. However, a previous study conducted in this prison reported that the prison population did not change over time (same rates of mental health treatment, of substance use and same socio-demographic profiles) [11]. Other factors related to prison life may also be included in future studies, such as time spent locked up in cells, unsafe cell conditions, bullying, or supportive relationships with the prison staff [15]. Indeed, the high prevalence rate of self-harm may also be due to the specific characteristics of the Champ-Dollon prison, namely the lack of freedom of movement, physical activities within the prison and other pro-health and pro-social activities. Indeed, these factors may reduce the negative impact of overcrowding on wellbeing and health indicators.

## 5. Conclusions

With their damaging effects on the health of PLD, overcrowding and turnover raise important human rights concerns. Reduction of prison overcrowding and turnover appears as critical to reduce self-harm in prison. Future preventive interventions should consider these important institutional risk factors along with individual risk factors to develop adapted strategies. Prison may be the opportunity to identify and initiate care for PLD at risk of self-harm [13].

## Figures and Tables

**Table 1:** Meta-regression of the effect of overcrowding and turnover on self-harm events.

Variables	Self-Harm	
	b	*p*-Value
Intercept	−0.778	<0.001
Overcrowding index	0.068	<0.001
Turnover ratio	1.266	<0.001

b values correspond the effect of each factor: for example, an increase of 1 on the overcrowding index corresponds to an increase of 0.068 on the prevalence estimate of self-harm.
